# Effects of transcranial direct current stimulation on brain cytokine levels in rats

**DOI:** 10.3389/fnins.2022.1069484

**Published:** 2022-12-23

**Authors:** Victoria T. Ethridge, Nathan M. Gargas, Martha J. Sonner, Raquel J. Moore, Shannon H. Romer, Candice Hatcher-Solis, Joyce G. Rohan

**Affiliations:** ^1^Naval Medical Research Unit Dayton (NAMRU-D), Wright-Patterson Air Force Base, Dayton, OH, United States; ^2^Odyssey Systems Consulting Group, Wakefield, MA, United States; ^3^Leidos, Reston, VA, United States; ^4^ICON, Hinckley, OH, United States; ^5^Infoscitex, Dayton, OH, United States; ^6^711th HPW/RHBCN, Wright-Patterson Air Force Base, Dayton, OH, United States

**Keywords:** tDCS, cytokines, inflammation, rats, plasticity

## Abstract

Transcranial direct current stimulation (tDCS) has shown therapeutic potential to mitigate symptoms of various neurological disorders. Studies from our group and others used rodent models to demonstrate that tDCS modulates synaptic plasticity. We previously showed that 30 min of 0.25 mA tDCS administered to rats induced significant enhancement in the synaptic plasticity of hippocampal neurons. It has also been shown that tDCS induces expression of proteins known to mediate synaptic plasticity. This increase in synaptic plasticity may underly the observed therapeutic benefits of tDCS. However, the anti-inflammatory benefits of tDCS have not been thoroughly elucidated. Here we report that three sessions of tDCS spaced 1–3 weeks apart can significantly reduce levels of several inflammatory cytokines in brains of healthy rats. Rats receiving tDCS experienced enhanced synaptic plasticity without detectable improvement in behavioral tests or significant changes in astrocyte activation. The tDCS-mediated reduction in inflammatory cytokine levels supports the potential use of tDCS as a countermeasure against inflammation and offers additional support for the hypothesis that cytokines contribute to the modulation of synaptic plasticity.

## Introduction

Transcranial direct current stimulation (tDCS) is a non-invasive method of brain stimulation with promising potential as a therapy or countermeasure against adverse neurological symptoms induced by traumatic brain injury (Clayton et al., 2016) and neurological diseases such as Alzheimer’s disease, Parkinson’s disease, stroke, epilepsy, and depression (Floel, 2014; An et al., 2017; Peanlikhit et al., 2017). Previous studies reported by us, and others revealed that tDCS enhances synaptic plasticity, including long-term potentiation (LTP) (Rohan et al., 2015, 2020; Podda et al., 2016; Strube et al., 2016; Kim and Han, 2017; Kim et al., 2017). We previously showed that a single 30 min tDCS session (of 0.1 or 0.25 mA of anodal or cathodal tDCS) induced a significant enhancement in LTP in the hippocampi of rats that could be measured 30 min and 24 h after the tDCS (Rohan et al., 2015, 2020). Administration of tDCS also induced detectable increases in brain derived neurotrophic factor (BDNF) levels as well as other proteins essential for neural plasticity (Podda et al., 2016; Kim et al., 2017).

The use of tDCS as a countermeasure against inflammation has not been clearly established. Studies using a rodent model of neuropathic pain and obesity demonstrated that tDCS can prevent increases in IL-1β, TNF-α and IL-10 triggered by chronic constriction injury in a neuropathic pain model or hypercaloric diet in a model for obesity (Cioato et al., 2016; de Oliveira et al., 2019). Recently, a study evaluating the effects of tDCS on inflammatory markers in naïve rats reported that a single session of 0.5 mA tDCS for 20 min resulted in a significant reduction in TNF-α levels in the brain at 30 min after tDCS, although this was not reflected at later timespoints (60 min, 120 min, 24 h) (Callai et al., 2022). However, that same study reported no significant changes in IL-10 levels at any of the time points tested (30, 60, 120 min and 24 h following tDCS). The study did not report measures of other cytokine levels.

To determine the effects of tDCS on inflammatory cytokines, we exposed male Sprague Dawley rats to three sessions of anodal tDCS at 0.25 mA for 30 min, each separated by 1–3 weeks. Here we show that following tDCS, LTP is enhanced without impacting behavioral performance. Additionally, we show that following tDCS, five out of nine inflammatory cytokines tested were significantly reduced without evidence of increased astrocyte activation. Altogether, these data support that tDCS has a therapeutic potential to reduce inflammation.

## Methods

### Animals

All animals were held and treated according to Wright Patterson Air Force Base (WPAFB) Institutional Animal Care and Use Committee (IACUC) and National Institutes of Health (NIH) guidelines. The study protocol was reviewed and approved by the WPAFB IACUC and was in compliance with all federal regulations governing the protection of animals and research. Male Sprague–Dawley rats (*Rattus norvegicus*) approximately 6 weeks of age weighing ∼150–200 grams at receipt were purchased from Charles River (Wilmington, MA, USA). Animals underwent acclimation for about 2 weeks in our animal facility. Animal rooms were maintained at a temperature and relative humidity in accordance with the recommendations of the NRC’s *Guide for the Care and Use of Laboratory Animals*, with approximately 15 complete air changes per hour, and a 12/12 h electronically controlled light/dark cycle. Food and water were always available except when the animals were undergoing surgery. Animals were singly housed following surgical procedures to preserve the welfare of the animals and integrity of the study by preserving the surgical site.

### Surgery

All rats underwent surgical procedures for placement of the electrode holder needed to perform tDCS. A circular 2.5 mm radius electrode was used for our specialized tDCS system so that only the electrode casing is implanted onto the scalp for connection with the tDCS electrode. Animals were anesthetized with isoflurane (Med-Vet International, Mettawa, IL, USA) using 5% induction, followed by 2–3% isoflurane to maintain anesthetic depth. Lidocaine was injected subcutaneously (s.c.) around the incision site on the skull. Buprenorphine was injected s.c. along the loose skin between the shoulder blades to allow fast post-operative analgesia. Gentamicin was also injected s.c. along the loose skin of the animal’s lower back. A 5 mm diameter, circular, head electrode casing (Tangible Solutions, Fairborn, OH, USA) was attached to the skull from 0 to −5 mm bregma. Luting dental cement (GC Fuji I, GC America Inc., Alsip, IL, USA) was applied to base of the head electrode casing and to the skull, followed by an acrylic dental cement (Sigma-Aldrich, St. Louis, MO, USA) to secure the electrode. Following surgery, rats were allowed to recover for 2 weeks before tDCS administration.

### Experimental timeline

**FIGURE 1:**
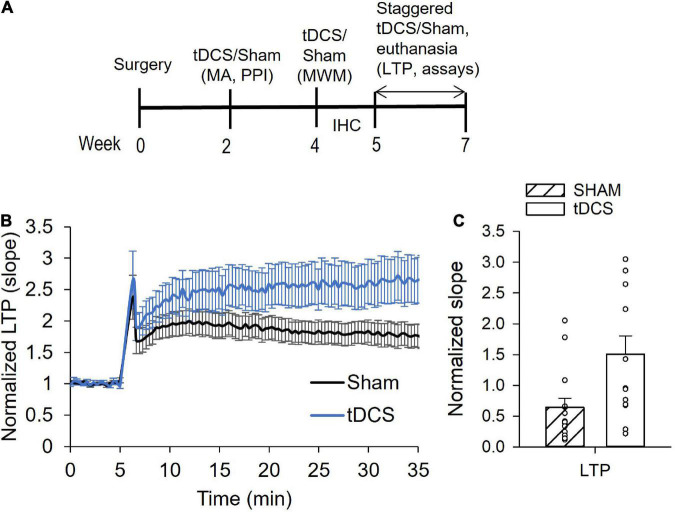
Effects of transcranial direct current stimulation (tDCS) on hippocampal long-term potentiation (LTP). **(A)** Diagram showing the experimental timeline. Rats underwent surgery and 3 rounds of tDCS before LTP and cytokine levels were measured. **(B)** Normalized slope data showing tDCS effect on LTP. Administration of tDCS resulted in enhanced LTP in the Shaffer collateral-CA1 synapses of rat hippocampus. **(C)** Bar graph showing significant increase in LTP from rats given 3 rounds of tDCS (*p* = 0.009, *n* = 15 slices sham, *n* = 13 slices tDCS, Mann Whitney).

The experimental timeline for tDCS and endpoints is shown in Figure 1A. A total of 30 rats were used in this study. Upon arrival, rats were randomized into the sham or tDCS groups. Three sham and three tDCS rats were randomly chosen to be used in the immunohistochemical study and the remaining 12 sham and 12 tDCS rats will be subjected to behavioral tests and used in cytokine assays and electrophysiological endpoints. The first round of tDCS/sham occurred 2 weeks following surgery for placement of the electrode holder. The second round of tDCS/sham was conducted 2 weeks following the first round. Administration of the third round of tDCS/sham was staggered to accommodate the electrophysiology recording schedule in which we recorded 2 rats per day (one tDCS, one sham). Thus, the third round of tDCS/sham was administered between 1 and 3 weeks after the second round of tDCS/sham. Rats used to assess astrocyte activation using immunohistochemistry and confocal microscopy were administered only one round of tDCS/sham 2 weeks following surgery. Behavioral tests were performed following one or two rounds of tDCS, for motor activity/acoustic startle reflex with prepulse inhibition and Morris water maze, respectively. For the electrophysiology and cytokine measurements, rats were given 3 rounds of tDCS or sham. Rats were euthanized 30 min following the 3rd tDCS session for the electrophysiology and biochemistry endpoints. For the immunohistochemistry endpoint, rats were euthanized 2–3 days following one tDCS session *via* exsanguination and transcardial perfusion fixation.

### Administration of tDCS

All rounds of tDCS were administered in unanesthetized and unrestrained rats. Conducting medium (SignaGel, Parker Laboratories, Fairfield, NJ, USA) was placed into the head casing prior to connecting the head electrode. The reference electrode (12 mm diameter, Tangible Solutions, Fairborn, OH, USA) was placed on the rat’s shaved chest with SignaGel as the conducting medium. Once the electrodes were in place, the animal was wrapped with a flexible cohesive bandage (PetFlex, Med-Vet, Mettawa, IL, USA) and placed into an open arena. Anodal tDCS was then applied at 0.25 mA using a constant-current stimulator (Magstim DCstimulator; Neuroconn, Ilmenau, Germany) for 30 min. The sham group was prepared the same way as the stimulation groups but did not receive any current. The first tDCS session was conducted just prior to motor activity and acoustic startle testing, the second round was conducted 2 weeks later and prior to the Morris water maze testing, and the third round occurred 1–3 weeks later and just prior to brain collection for electrophysiology and cytokine measurements.

### Behavioral tests

We conducted motor activity, acoustic startle reflex with prepulse inhibition and Morris water maze tests following tDCS or sham administration. Methodologies for these behavioral tests were performed as previously described (Rohan et al., 2018; Gargas et al., 2021a,b). Rats were subjected to an open field test to assess motor activity approximately 30 min following the first session of tDCS (0.25 mA for 30 min). The next day, rats were subjected to the acoustic startle test with prepulse inhibition using San Diego Instruments sound and light attenuation chambers and the provided computer software (SR-LAB). Two weeks after the first tDCS session, rats were subjected to the second round of tDCS, and Morris water maze testing began the next day. More information on behavioral procedures can be found in the Supplementary methods. All behavioral testing were conducted by investigators blinded to the groups.

### Long-term potentiation (LTP)

The third tDCS session was administered 1–3 weeks after the second tDCS session and approximately 30 min prior to brain collection for electrophysiology and cytokine measurements. LTP data were obtained using a microelectrode array system [AlphaMed MED64 Quad II systems (Automate, Berkeley, CA, USA)] and is described in detail elsewhere (Rohan et al., 2015, 2020; Gargas et al., 2021b). LTP was induced by delivering 3 trains of theta burst stimulation (TBS), consisting of 10 repeats of 4 high frequency stimulations (100 Hz) every 200 ms to the Schaffer collateral region. Evoked responses in the forms of fEPSP and population spikes were monitored at 12 s intervals for at least 20 min following LTP induction. Percent potentiation was calculated by computing the percent difference in fEPSP slope at 30 min following LTP induction by TBS from baseline. Averages of 10 data points for each were calculated to obtain baseline and LTP values. Acquisition of electrophysiological recordings were conducted in a blinded fashion.

Analyses of electrophysiology data were performed using Mobius software. Microsoft Excel and SigmaPlot v.13 were used to plot analyzed data and for statistical assessments. The slope of the downward (negative, inward) field potentials (fEPSP) were used to calculate degree of evoked response. Slope calculations were performed using Mobius software using its “Slope1040LinearFit” measurement option. Neuronal activity was recorded from 10 to 15 microelectrodes within the MED64 probe chamber. For any given slice, data obtained from multiple microelectrodes covering one defined brain region (e.g., CA1) were averaged together to yield the response value for that particular slice. Statistical analyses were then performed on the data obtained from the different brain slices. Data are represented as means with the standard error of the mean (SEM) and were statistically compared using the two-tailed, unpaired *t*-test. A calculated *p*-value of less than 0.05 is considered significantly different. All quantitation and statistical analysis as well as graphs were generated using Microsoft Excel and SigmaPlot v.13.

### Cytokine analyses

Cytokine levels were measured in plasma and brain homogenates. Brain samples were homogenized in 1 mL of PBS per 0.5 g of tissue. Samples were homogenized with a tissue tearor (Bio Spec Products, Inc., Bartlesville, OK, USA). Following a minimum of two freeze/thaw cycles, the homogenates were centrifuged. The supernatant was decanted and used for analysis. Brain homogenate samples were diluted, as needed. Multiple inflammatory cytokines (IFN-γ, IL-1β, IL-4, IL-5, IL-6, KC/GRO, IL-10, IL-13, and TNF-α) were measured using a multi-spot cytokine assay system (Meso Scale Discovery, Rockville, MD, USA). Investigators conducting the assays were initially blinded to the groups. Samples were analyzed per manufacturer supplied protocols. Cytokine measurements were performed in duplicates and data with a coefficient of variation (CV) greater than 25 were omitted. Statistical comparison between the sham and tDCS groups were conducted using the two-tailed, unpaired *t*-test.

### Immunohistochemistry and confocal microscopy to assess astrocyte activation

To prepare tissue for immunohistochemistry, rats were first deeply anesthetized with 100 mg/kg sodium pentobarbital *via* intraperitoneal injection. Rats were then transcardially perfused with 4% paraformaldehyde in 0.1 M phosphate buffer at pH 7.3. The brains were removed, hemisected and placed in 4% paraformaldehyde overnight then cryoprotected in 15% sucrose. Coronal brain slices (approximately 70 μm) were obtained on a cryostat (Leica Biosystems CM1850, Nußloch, Germany) at the level of the hippocampus and floating sections were immunostained in 6-well plates. Following incubation in Normal Horse Serum Blocking Solution (Vector Laboratories catalogue # S-2000-20, Burlingame, CA, USA) astrocytes were labeled with mouse anti-glial fibrillary acidic protein (GFAP; BD Pharmingen catalogue # 556330, San Diego, CA, USA). Immunoreactivity was detected with donkey-anti-mouse secondary antibody conjugated to Cy3 fluorophore (Jackson Immuno #715-165-150, West Grove, PA, USA). Green fluorescent Nissl staining was used to label neurons (Invitrogen catalogue # N21480, Waltham, MA, USA). Tissue was mounted in Vectashield containing 4′,6-diamidino-2-phenylindole (DAPI) to label nuclei (Vector Laboratories catalogue # D3571, Burlingame, CA, USA). Microscopy images were obtained on a Leica SP8 confocal microscope (Leica Microsystems, Wetzlar, Germany) with a 20× objective at 1.0 μm *z*-steps. Multiple image stacks were collected to cover the entirety of the unilateral hippocampus and merged together into a single image in LasX software (Leica Microsystems, Wetzlar, Germany). Quantification of the merged microscopy images was performed in Image Pro Software (Media Cybernetics, Bethesda, MD, USA) by investigators blinded to the groups.

To quantify the number of astrocytes, a 400 by 400-pixel (303.2 μm by 303.2 μm) grid overlay was applied to a single optical section of the confocal image stacks. Ten boxes were randomly selected within the grid for analysis. Astrocytes were identified by the GFAP positive immunoreactivity and counted at the nuclei, revealed by DAPI staining, to ensure a single count per cell. For each animal, a total of three single optical sections were analyzed. To quantify the intensity of the GFAP immunolabeling, a region of interest was designated to encompass the entirety of the unilateral hippocampus region in a single optical image section containing only GFAP immunoreactivity. Integrated optical density was used to summate the intensities of all pixels contained within the region of interest. Minimum, maximum, and mean intensities were also collected. For each animal, three hippocampus tissue sections were quantified and averaged per animal.

## Results

A total of 30 rats were used in this study. All rats underwent surgery to implant tDCS probe holder. Half of the rats were placed in the sham group and the other half in the tDCS group. A total of 12 sham and 12 tDCS rats were subjected to motor activity and acoustic startle reflex with prepulse inhibition. A total of 11 sham rats and 10 tDCS rats were subjected to Morris water maze tests. Three rats were not subjected to Morris water maze test due to the slightly loose dental cement that could increase the risk of infection when submerged in water. Plasma and half of brain from all 12 sham and 12 tDCS rats were collected and used for biochemical measurements. However, only cytokine data with less than 25 coefficient of variation (CV) and within the standard curves were used in the data analysis. Electrophysiology recordings were conducted on 9 sham and 9 tDCS rats, at 1–2 slices per rat.

### Anodal tDCS enhanced LTP without detectable improvement in behavioral performance

Previously, we reported that male and female rats administered 30 min of tDCS at 0.25 mA intensity had augmented synaptic plasticity (Rohan et al., 2015; Gargas et al., 2021a). Synaptic plasticity was assessed by measuring long-term potentiation (LTP) in Schaffer collateral-CA1 synapses of the hippocampus. Enhanced LTP was detected 30 min and 24 h following tDCS (Rohan et al., 2015). Another study reported that the tDCS-induced enhancement of plasticity is long lasting and can in fact still be detected 1 week following administration of tDCS (Podda et al., 2016). Here, we performed three rounds of tDCS (0.25 mA, 30 min), with the first two rounds spaced 2 weeks apart and the third round 1–3 weeks thereafter, after which the brain was extracted for electrophysiology and cytokine measurements (Figure 1A).

We did not observe any statistically significant improvement in behavioral performance following 30 min of tDCS at 0.25 mA (Supplementary Data). Administration of tDCS did not induce significant changes in motor activity parameters with the exception of total rears (Supplementary Table 1). There was a statistically significant reduction in total rears resulting from tDCS rats compared to sham rats, from an average of 64.67 ± 5.6 to 47.17 ± 3.6 [*t*(22) = 2.633, *p* = 0.05, *n* = 12]. To assess the effects of tDCS on sensorimotor gating, we subjected rats to the acoustic startle reflex test with prepulse inhibition. Impaired modulation of the acoustic startle reflex response has been associated with impaired sensorimotor gating observed in patients suffering from schizophrenia, ADHD and autism (Dawson et al., 1993; Li et al., 2009; Kohl et al., 2014). Acoustic startle reflex with prepulse inhibition showed a slight increase in startle attenuation with tDCS that was not statistically significant [*t*(22) = 1.745, *p* = 0.09, *n* = 12] (Supplementary Figure 1). To evaluate the effects of tDCS on spatial learning and memory, we subjected sham and tDCS rats to Morris water maze test. Morris water maze test revealed statistically significant increases in latency to target with rats administered tDCS during the early learning days (Day 1–3) but the increases were not statistically significant on Day 4 and 5 (Supplementary Figure 2A). There was no effect of tDCS on memory as rats administered tDCS had similar latency, distance from target and speed as control rats on the memory probe test which occurred on Day 6 (Supplementary Figures 2B–D).

A total of 9 sham and 9 tDCS rats were used for LTP recordings at 1–2 slices per rat. Typical amplitudes of evoked responses were 200–1,500 μV. Evoked responses of less than 50 μV were omitted from data analysis. There was a significant enhancement of LTP in hippocampal neurons (at the Schaffer collateral-CA1 synapse) in rats administered tDCS compared to control rats (sham) (Figures 1B, C). The average slope of responses following LTP induction increased over two-fold in rats that were administered tDCS. *T*-test analysis yielded a *p-*value of 0.01 (*n* = 15 slices sham, 13 slices tDCS). However, our LTP data failed both normality and equal variances test, using the Shapiro–Wilk and the Brown-Forsythe tests, respectively. Thus, Mann Whitney analysis of LTP data was also performed, yielding a *p-*value of 0.009. These data are consistent with our previous finding in which rats displayed enhanced LTP 30 min and 24 h following 30 min of 0.25 mA tDCS (Rohan et al., 2015).

### Anodal tDCS reduced brain cytokine levels in male rats

**FIGURE 2:**
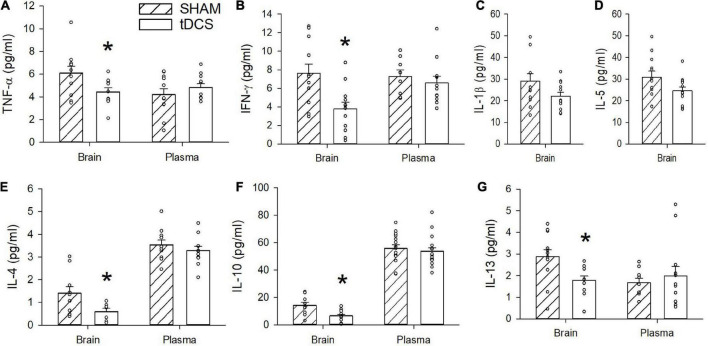
Effects of transcranial direct current stimulation (tDCS) on brain and plasma cytokine levels. **(A)** TNF-α was significantly reduced in the brains of rats stimulated with tDCS (*p = 0.03, *n* = 11, *t*-test). No significant difference was observed in plasma levels of TNF-α (*p* > 0.05). **(B)** IFN-γ was significantly reduced in the brains of rats stimulated with tDCS (*p = 0.004, *n* = 12, *t*-test). No significant difference was observed in plasma levels of IFN-γ (*p* > 0.05). **(C)** A non-significant decrease in IL-1β level was detected in brains of rats administered tDCS with *p* = 0.07 (*n* = 11 sham, 12 tDCS, *t*-test). Plasma levels of IL-1 β were below the detection limit. **(D)** A non-significant decrease in IL-5 was detected in brains of rats administered tDCS with *p* = 0.08 (*n* = 12, *t*-test). Plasma levels of IL-5 were below the detection limit. **(E)** IL-4 was significantly reduced in brains of rats stimulated with tDCS (*p = 0.04, *n* = 10 sham, 7 tDCS, *t*-test). No significant difference in plasma level of IL-4 (*p* > 0.05). **(F)** IL-10 was significantly reduced in brains of rats administered tDCS (*p = 0.005, *n* = 9 sham, 12 tDCS, *t*-test). Plasma level of IL-10 was not significantly different (*p* > 0.05). **(G)** IL-13 was significantly reduced in brains of rats stimulated with tDCS (*p = 0.01, *n* = 12 sham, 11 tDCS, *t*-test). There was no significant difference in IL-13 in the plasma (*p* > 0.05).

We measured levels of IFN-γ, IL-1β, IL-4, IL-5, IL-6, KC/GRO, IL-10, IL-13, and TNF-α in plasma and in homogenized brain from rats administered three rounds of tDCS and sham-exposed rats. We found no significant changes in any of the measured cytokines in the plasma resulting from tDCS administrations. However, we did detect statistically significant reduction in levels of IFN-γ, TNF-α, IL-4, IL-10, and IL-13 in homogenized brains from rats subjected to tDCS (Figure 2). Cytokine levels were measured in duplicates and samples with a coefficient of variation (CV) greater than 25 were omitted. Some samples fell under the detection limit and thus were also omitted. The average brain level of IFN-γ from rats administered tDCS was reduced by 51% with [*t*(22) = 3.192, *p* = 0.004 (*n* = 12)]. The average brain level of TNF-α was reduced by 27% with [*t*(20) = 2.339, *p* = 0.03 (*n* = 11)]. The average brain level of IL-4 was reduced by 58% with [*t*(15) = 2.205, *p* = 0.04 (*n* = 10 sham, 7 tDCS)]. The average brain level of IL-10 was reduced by 54% with [*t*(19) = 3.144, *p* = 0.005 (*n* = 9 sham, 12 tDCS)]. The average brain level of IL-13 was reduced by 38% with [*t*(21) = 2.773, *p* = 0.01 (*n* = 12 sham, 11 tDCS)]. Administration of tDCS induced only minor decreases in brain levels of IL-1β [*t*(21) = 1.904, *p* = 0.07, *n* = 11 sham, 12 tDCS] and IL-5 [*t*(22) = 1.819, *p* = 0.08, *n* = 12]. No changes in brain IL-6 and KC/GRO levels were observed after tDCS (*p* = 0.9, 0.4, respectively, with IL-6 *n* = 12 and KC/GRO *n* = 11 sham, *n* = 12 tDCS). All data were tested for normality and equal variances using the Shapiro–Wilk and the Brown-Forsythe tests, respectively. All cytokine data passed both normality and equal variances with the exception of brain IL-10 cytokine, which failed the equal variances test and brain IL-6, which failed the normality test. We conducted the non-parametric Mann Whitney test for the brain IL-10 data and obtained *p* = 0.012. Mann Whitney test of the plasma IL-6 levels still revealed a non-significant effect at *p* = 0.47.

The brain cytokine levels were measured 30 min following the 3rd tDCS session, which was conducted at 1–3 weeks following the 2nd tDCS session. The reason behind the stagger administration of the 3rd tDCS was because electrophysiology was conducted on half of the brain from the same animals, and we can only record from a limited number of rats per day. Thus, two rats were euthanized per day at 30 min following the 3rd tDCS administration, to accommodate the electrophysiology recording. To determine whether the cytokine measurements depended on the time interval between the 2nd and 3rd tDCS sessions, we plotted levels of various cytokines as a function of interval days between the 2nd and 3rd tDCS session (Supplementary Figure 3). We found no significant correlation between when the 3rd tDCS was administered and brain cytokine levels of TNF-α, IFN-γ, IL-1β, IL-5, IL-4, IL-10, or IL-13 (Supplementary Figure 3). Therefore, we hypothesize that the effects on brain cytokine levels was primarily due to the 3rd tDCS session and independent of the first two tDCS sessions.

### Anodal tDCS did not induce significant effects on astrocyte activation in the hippocampus

**FIGURE 3:**
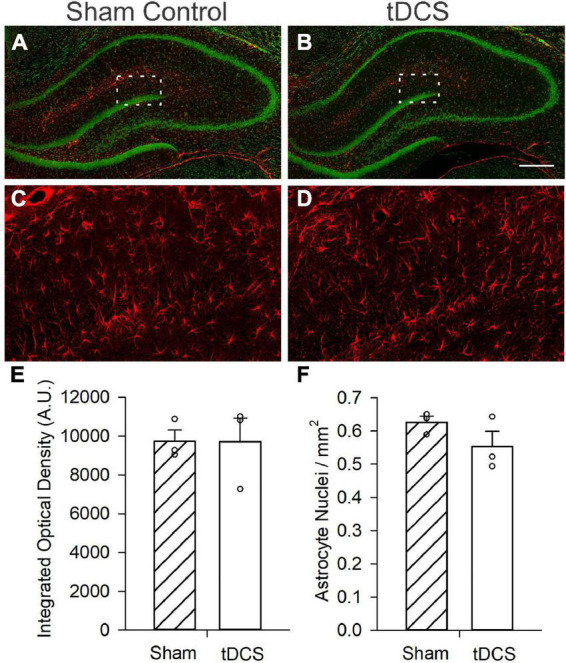
Effects of transcranial direct current stimulation (tDCS) on astrocyte activation. Micrograph images from small confocal stacks (*z* = 9 × 1 μm steps) with astrocytes revealed with GFAP immunoreactivity (Red) and neurons revealed with green fluorescent Nissl staining. **(A)** Image of hippocampus slice from a control (sham) rat. **(B)** Image of hippocampus slice from a rat administered one round of tDCS. Scale bar = 400 μm **(C)** Enlarged image from a control (sham) rat showing astrocytes. Area enlarged is indicated as the white box in A. **(D)** Enlarged image from a rat administered one round of tDCS showing astrocytes. Area enlarged is indicated as the white box in B. **(E)** Bar graph showing no significant effect of tDCS on integrated optical density (*p* = 0.985, *n* = 3 rats, *t*-test) **(F)** Bar graph showing a minor decrease in astrocyte nuclei per mm^2^, effect was not statistically significant (*p* = 0.211, *n* = 3 rats, 3 slices per rat, *t*-test).

A total of 3 sham and 3 tDCS rats at 3 slices per rat were used to assess effects on astrocyte activation. It has been suggested that effects of anodal tDCS on astrocyte activation may play a mechanistic role in its modulating effect on synaptic plasticity and potential therapeutic effect on depression (Monai and Hirase, 2016, 2018). Glial fibrillary acidic protein (GFAP) is an intermediate filament protein expressed by astrocytes in the central nervous system (CNS). GFAP levels have been used as a marker of CNS damage. Increases in GFAP immunoreactivity have been associated with increased reactive astrocytes and neuroinflammation. Here we found that administration of a single 30 min 0.25 mA tDCS did not induce a significant change in GFAP immunoreactivity patterns in the hippocampi of rats (Figure 3). There was a slight reduction (12% decrease in average value) in the number of GFAP-positive astrocytes from hippocampi of rats administered tDCS compared to controls, but this reduction was not statistically significant (*p* = 0.2, *n* = 3). Our data is consistent with a recent study in which tDCS at 0.5 mA for 20 min did not induce significant changes in GFAP levels in rat cerebral cortex (Callai et al., 2022). All data passed normality and equal variances test.

## Discussion

Overall, our data show that tDCS administration at a level that enhances synaptic plasticity (LTP) does not activate astrocytes and can reduce inflammatory cytokine levels in the brain, supporting tDCS as a potential inflammatory therapy. Cioato et al. (2016) reported that 20 min of 0.5 mA tDCS for eight consecutive days in a rat model of neuropathic pain caused by chronic constriction injury of the sciatic nerve prevented the increase in CNS levels of IL-1β and IL-10. A subsequent study from the same group demonstrated that tDCS prevented the increase in IL-1β and TNF-α caused by a hypercaloric diet in rats (de Oliveira et al., 2019). A more recent study showed that tDCS given to naïve rats induced significant reduction of TNF-α in the brain (Callai et al., 2022). The intensity of tDCS used in these previous studies was twice the tDCS intensity we used here. Our study used a tDCS intensity level that is more comparable to the level used in human subjects and we demonstrated that this level was sufficient to induce detectable LTP enhancement in hippocampal neurons (Figures 1B, C). However, behavioral tests did not reveal significant improvement in motor performance or cognition (Supplementary material). We detected a small reduction in total rears by rats administered tDCS compared to control rats. Although reduced rearing has been correlated with stress and/or anxiety, the reduction in rearing seen here (27%) is significantly less than that reported in stressed rats (reduction by ∼67%) (Sturman et al., 2018). Furthermore, stressed or anxious rats often display decreased time in the center whereas tDCS rats spent nearly identical time in the center as sham rats. The increased latency in the early learning days of Morris water maze test may indicate a hyperactive state with tDCS that did not manifest as impaired learning since latency was no longer significantly different during the last 2 days of learning, and memory performance was identical in tDCS administered and control rats. This is in contrast to an earlier study in which a single tDCS session decreased the latency in finding the target in mice (Podda et al., 2016). The disparity in result can be attributed to potential species differences and/or differences in our water maze procedures. Other past studies using rats that demonstrated significant positive effects of tDCS on cognitive function had employed rats that were intentionally impaired to model various neurological disorders, such as Alzheimer’s disease (Yu et al., 2015; Yang et al., 2019). Thus, the lack of significant improvement seen in our study may also be attributed to a ceiling effect in fully functional animals, in which the Morris water maze task performed by the control rats likely reached the highest possible score such that tDCS could not further improve the performance score.

Our cytokine measurements corroborate the observation of decreased TNF-α levels in the brains of naïve rats. Furthermore, our data demonstrate that tDCS can cause significant reductions in additional inflammatory cytokines (IFN-γ, IL-4, IL-10, and IL-13) and non-significant decreases in IL-1β and IL-5 with *p-*values of 0.07 and 0.08, respectively. The physiological significance of decreased inflammatory cytokines in the brain requires additional studies. IFN-γ,TNF-α, and IL-1β are pro-inflammatory cytokines and their increases have been associated with astrocyte and microglia activation in CNS (Wong et al., 1997; Lively and Schlichter, 2018). Although IL-4 is known for its anti-inflammatory effects, it can also contribute to inflammation by activating macrophages (Luzina et al., 2012). Previous work also demonstrated that dysregulation of IL-10, another cytokine known for its anti-inflammatory properties, can contribute to neurodegeneration (Iyer and Cheng, 2012; Porro et al., 2020). Furthermore, IL-13 has been associated with loss of dopaminergic neurons during inflammation (Mori et al., 2016).

Cytokines in the CNS play diverse roles, but those roles and mechanisms have not been clearly identified. In addition to being associated with inflammatory processes, published studies suggests that cytokines have other physiological roles in brain function. Several pro-inflammatory cytokines such as IFN-γ,TNF-α, and IL-1β can modulate neuronal excitability and brain circuitry *via* their effects on various neurotransmitter systems (Galic et al., 2012; Miller et al., 2013). In addition to its effects on microglia and macrophages, IL-4 also mediates the essential effects of T cells on brain function (Gadani et al., 2012). Furthermore, a recent study reported that several cytokines including IL-1β, IL-4, IL-10, IL-13, and TNFα are upregulated with age in the CNS, demonstrating the significance of cytokines in brain development and aging (Porcher et al., 2021). Upregulation of inflammatory cytokines in the brain has been associated with adverse neurological outcome and has been correlated with various neurological diseases especially multiple sclerosis (MS) and Alzheimer’s disease (AD) (Steinman, 2008). Additionally, systemic inflammation induced by viral or bacterial infection, can trigger massive neuroinflammation in the brain known as cytokine storm (Mishra and Banerjea, 2020). There is also growing evidence associating high brain cytokine levels with psychiatric disorders such as dementia and delirium (Yarlagadda et al., 2009). Thus, the reduction in cytokines observed with tDCS can potentially be beneficial in alleviating neurological or psychiatric symptoms, mitigating the progression of neurological diseases such as MS or AD, or reducing the likelihood of a cytokine storm following systemic infections.

Although more research is needed to further characterize the various functions of cytokines in the CNS, data presented here demonstrate that tDCS is capable of altering the levels of several cytokines in the brain. Such capability can be one mechanism by which tDCS exerts its beneficial effect in various rodent models of neurological diseases and in patients suffering from various neurological ailments.

## Data availability statement

The original contributions presented in this study are included in the article/Supplementary material, further inquiries can be directed to the corresponding author.

## Ethics statement

This animal study was reviewed and approved by Wright-Patterson Air Force Base (WPAFB) Institute of Research Institutional Animal Care and Use Committee (IACUC).

## Author contributions

JR planned and designed the overall project. VE and NG collected and analyzed the electrophysiology and biochemical data. SR and MS conducted and provided the immunohistochemistry and imaging data. CH-S designed the tDCS parameters. CH-S and RM performed the tDCS surgeries and stimulations. VE and JR drafted the first manuscript. All authors participated in subsequent revisions.

## Author Disclaimer

The views expressed in this presentation are those of the authors and do not necessarily reflect the official policy or position of the Department of the Navy, Department of Defense, nor the U.S. Government. The study protocol was reviewed and approved by the Wright-Patterson Air Force Base (WPAFB) Institute of Research Institutional Animal Care and Use Committee (IACUC) incompliance with all applicable Federal regulations governing the protection of animals in research and conducted in an AAALAC-accredited facility.

The authors are federal/contracted employees of the U.S. Government. This work was prepared as part of our official duties. Title 17 U.S.C. §105 provides that “Copyright protection under this title is not available for any work of the United States Government.” Title 17 U.S.C. §101 defines U.S. Government work as work prepared by a military service member or employee of the U.S. Government as part of that person’s official duties.
