# Supplementary material for: Effects of transcranial direct current stimulation on brain cytokine levels in rats
Source: Front Neurosci. 2022 Dec 23;16:1069484. doi: 10.3389/fnins.2022.1069484 (PMC9822516; doi:10.3389/fnins.2022.1069484)
Supplement: Supplementary file 1 [file Data_Sheet_1.pdf]

SUPPLEMENTARY MATERIALS

Supplementary Table 1. There were no statistically significant changes in the motor activity parameters (means ± sem) resulting from the first round of tDCS, with the exception of total rears. There was a statistically significant decrease in total rears from rats administered tDCS compared to control rats (Sham).

	Actual Distance (cm)	Actual Speed (cm/sec)	Activity Time (sec)	Total beam breaks	% in Center	Fine Total	Ambulation Total	Total Rears
**Sham**	21947 ± 1812	36.12 ± 1.21	956.67 ± 64.4	1701.67 ± 146.8	54.93 ± 1.10	201.08 ± 6.48	3453.92 ± 270.7	64.67 ± 5.6
**tDCS**	19737 ± 1101	38.40 ± 1.14	881.94 ± 30.3	1486.08 ± 119.6	54.15 ± 1.30	199.83 ± 9.13	3060.83 ± 162.3	47.17 ± 3.6* (*p* = 0.02)

*p = 0.05, t-test, t(22) = 2.633, n = 12.

Supplementary Figure 1. Effect of tDCS on acoustic startle reflex with prepulse inhibition. Data presented as means ± sem. There was a slight increase in percent startle attenuation in rats administered tDCS compared to control rats (Sham) that was not statistically significant (t(22) = 1.745, p= 0.09, n= 12).

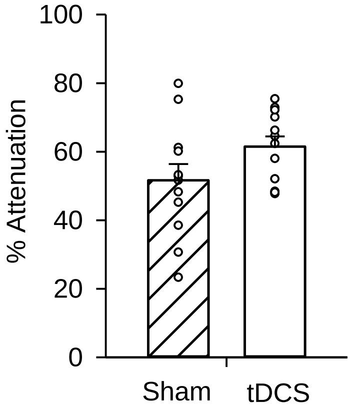

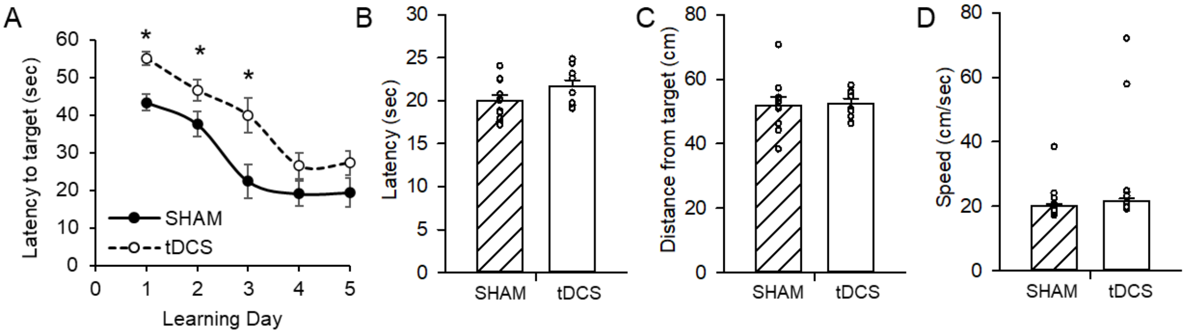

Supplementary Figure 2. Effects of tDCS on Morris water maze performance. Data presented as means ± sem. (**A**) There was an increase in latency to target during the learning phase of Morris water maze test in rats administered tDCS compared to control rats (Sham). The increase was statistically significant during learning day 1 (*p= 0.001), 2 (*p= 0.05), and 3 (*p= 0.01) but became non-significant on learning day 4 (p= 0.13) and 5 (p= 0.14). (**B-D**) There was no significant effect of tDCS on memory. Latency in reaching the target location (**B**), distance from target location (**C**) and speed (**D**) were similar in both Sham and tDCS rats, p> 0.05, n= 11 Sham, 10 tDCS (on Day 6).

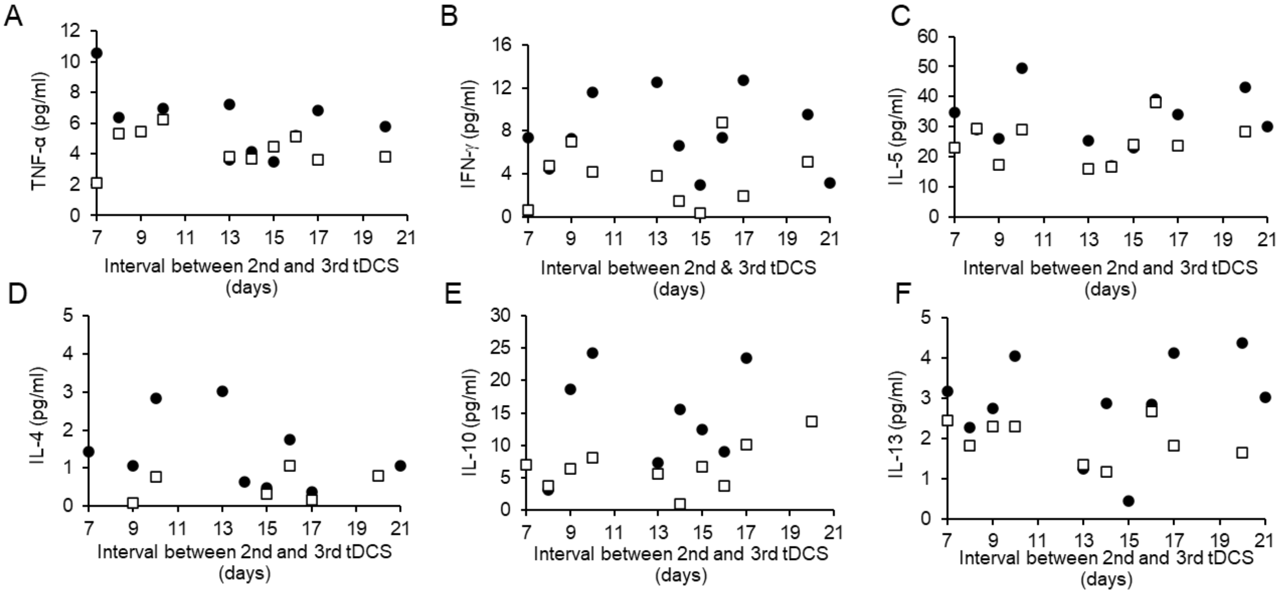

Supplementary Figure 3. Cytokine levels as a function of interval days between the 2nd and 3rd administration of tDCS. There was no correlation between the interval time and effects on brain cytokine levels of (**A**) TNF-α, (**B**) IFN-γ, (**C**) IL-5, (**D**) IL-4, (**E**) IL-10, (**F**) IL-13.

Supplementary Methods

Motor activity

Rats were placed in an open field to assess motor activity levels. Animals were placed into 40 cm by 40 cm open field activity chambers and allowed to move freely. Motor activities were measured using an automated photobeam data collection system (San Diego Instruments, Inc). Horizontal activity and vertical rears were scored using two separate sets of photobeams. Photobeams recorded both fine movements, reported as one broken photobeam, and ambulation, reported as two broken photobeams in sequence. Endpoints included total distance traveled in centimeters, animal speed in centimeters per second, time spent in center versus perimeter of the open field, and number of rears on hind limbs. Each test session lasted 30 minutes and motor activity habituation was determined over six blocks of five-minute intervals.

Acoustic startle reflex with prepulse inhibition

Acoustic startle test with prepulse inhibition was conducted using San Diego Instruments sound and light attenuation chambers and the provided computer software (SR-LAB). Animals received a number of discreet auditory startle sessions consisting of a series of 115 dB tones. Startle response and habituation were measured by the magnitude and latency of the startle response. Responses induced by startle tones alone were compared to those induced by startle tones that were preceded by a less intense prepulse tone (75 dB) to assess sensorimotor gating.

Morris water maze:

The Morris water maze test is a neurobehavioral test used to evaluate visuospatial learning, spatial navigation, and short-term as well as long-term spatial memory in rodents. The procedure consists of learning and memory components. For the learning phase, rats were given 4 training trials per day over a 5-day period. During these learning days, the animal was placed into a 7-foot diameter cylindrical

dark plastic tank filled with water. The temperature of the water was set at 19-25°C. A hidden escape platform (10 cm^2) was anchored to the floor of the tank but submerged 1 inch below the surface of the water. Four virtual quadrants were created inside of the tank using the SMART tracking software from San Diego Instruments. On the outside of the tank, there were 4 walls/screens with four large distinct shapes to serve as visual cues for location of the hidden escape platform. The animal will initially be placed facing the wall in one of the four external cue-based quadrants. Rats were allowed to swim until reaching the escape platform or until reaching the previously set maximum time of 60 seconds. Once the animal found the platform, the rat was given approximately 15 seconds to familiarize itself with its location. If an animal failed to locate the platform within 60 seconds, it was led to it and also given approximately 15 seconds for familiarization. Rats were subjected to 4 training trials per day with an inter-trial interval of 15 seconds between trials. Training will occur over a 5-day period. Learning parameters included latency to target (hidden platform), speed, and total distance traveled. The memory phase of the Morris water maze test is referred to as the probe trial and occurred approximately 24 hours from the last training run, on the 6th day. During the probe trials, the platform was removed, and the animal was placed diagonally across from the platform quadrant. The animal was allowed to swim for 60 seconds. Memory parameters included time spent in the target quadrant, distance from target, and total swimming distance.
